# Identification of a Prognostic Signature Based on Tumor-Infiltrating B Lymphocyte mRNA in Head and Neck Squamous Cell Carcinoma

**DOI:** 10.1155/jimr/9375885

**Published:** 2025-03-19

**Authors:** Mingjun Zhang, Qi Sun, Yisong Yao, Xi Chen, Jiaxuan Li, Ting Yuan, Yakui Mou, Yumei Li, Xicheng Song

**Affiliations:** ^1^Qingdao Medical College, Qingdao University, Qingdao 266000, China; ^2^Department of Otorhinolaryngology Head and Neck Surgery, Yantai Yuhuangding Hospital, Yantai 264000, China; ^3^Shandong Provincial Clinical Research Center for Otorhinolaryngologic Diseases, Yantai Yuhuangding Hospital, Yantai 264000, China; ^4^Shandong Provincial Key Laboratory of Neuroimmune Interaction and Regulation, Yantai Yuhuangding Hospital, Yantai 264000, China; ^5^Yantai Key Laboratory of Otorhinolaryngologic Diseases, Yantai Yuhuangding Hospital, Yantai 264000, China

**Keywords:** head and neck squamous cell carcinoma, immune cell infiltration, messenger RNA, prognostic signature, tumor-infiltrating B lymphocytes

## Abstract

**Introduction:** Tumor-infiltrating B cells (TILBs) are an important part of the immune response during tumor regulation. However, the significance of B cells in immunotherapy has not been fully determined.

**Methods:** In this study, highly expressed genes in B cells were obtained by comparing the gene expression in B cells with that in other immune cells and were named TILB-mRNAs. Among them, those genes expressed in patients with head and neck squamous cell carcinoma (HNSCC) identified in The Cancer Genome Atlas (TCGA) and Gene Expression Omnibus (GEO) atlas were employed to screen for genes associated with HNSCC prognosis using univariate Cox analysis, least absolute shrinkage and selection operator (LASSO) regression analysis, and a TILB-related signature was constructed to predict patient prognostic risk using multivariate Cox regression analyses.

**Results:** The constructed TILB-related signature, which comprised seven mRNAs (*ZNF439*, *KMO*, *KDM5D*, *IFT57*, *HDAC9*, *GSAP*, and *CCR*7), was verified to have a good ability to predict the prognosis of patients with HNSCC using three independent validation datasets from GEO, and the predictive ability was not affected by other factors. The signature reflected the state of immune cell infiltration in tumor tissue, especially B cells, patients with higher risk scores (RSs) had fewer infiltrating immune cells in their tumors, especially B cells. The gene expression of the TILB-related signature was also verified in TILBs from HNSCC using single-cell analysis, revealing that TILB-related marker genes were differentially expressed in different GB cell subsets.

**Discussion:** This study provides risk assessment and outcome prediction for patients with HNSCC and provides potential targets for immunotherapy of HNSCC.

## 1. Introduction

Head and neck squamous cell carcinoma (HNSCC), derived from the mucosal epithelium in the oral cavity, pharynx, and larynx, accounts for 90% of head and neck cancers. There are over 800,000 new HNSCC cases per year, resulting in more than 500,000 deaths annually [1]. The burden of HNSCC varies across countries and regions and generally correlates with exposure to tobacco-derived carcinogens and excessive alcohol consumption [2]. Despite improvements in methods of diagnosis and treatment, the 5-year survival rate and overall survival (OS) remain unsatisfactory [3]. Immunotherapy has become an important treatment for HNSCC, especially for recurrent and metastatic disease [4]. However, only a minority of patients benefit from immunotherapy. Studies have reported that the response to immunotherapy is associated with tumor-infiltrating lymphocytes (TILs) in the tumor microenvironment, with increased TIL levels predicting a good immunotherapy response [5].

Tumor-infiltrating B cells (TILBs) are an important part of the immune response [6] and can inhibit tumor progression, enhance the T cell response by acting as antigenpresenting cells, and promote the formation of a tertiary lymphoid structure (TLS) [7]. Ruffin et al. [8] reported that increased numbers of TILBs in the germinal center (GC) contributed to better OS in patients with human papillomavirus (HPV)-positive HNSCC. Currently, B cell-related pathways are believed to represent novel targets for immunotherapy [9]. Tumor immune cell infiltration is usually measured through staining of pathological sections under a microscope, followed by visual assessment; however, this method is subject to bias and variability. Recently, gene expression markers have been identified in TILB-related transcriptional signatures and have been used for quantitative assessment and prognostic stratification of TILBs [10, 11]. TILB-related signatures have been reported to have good predictive performance in bladder cancer and non-small cell lung cancer [12, 13]. Thus, TILB-related signatures might also have good predictive power in HNSCC; however, they have not yet been studied in HNSCC.

In this study, we collected raw microarray data for 19 types of immune cells in multiple data sets from the Gene Expression Omnibus (GEO) database and carried out background correction, log2 transformation, and quantile normalization on the raw data. Highly expressed genes in B cells were obtained by comparing gene expression between B cells and other immune cells, which were named TILB-mRNAs. Among them, genes expressed in patients with HNSCC in datasets from The Cancer Genome Atlas (TCGA) and GEO were used to construct a prognostic signature for HNSCC using univariate Cox analysis and least absolute shrinkage and selection operator (LASSO) regression analysis. A TILB-related signature was then constructed to predict patient prognostic risk using multivariate Cox regression analyses. The results showed that the TILB-related signature had a good ability to predict the prognosis of patients with HNSCC and reflected the state of immune cell infiltration in the tumor tissue, especially B cells. The gene expression of the TILB-related signature in B cells was verified to regulate HNSCC through single-cell analysis. This study provides risk assessment and outcome prediction for patients with HNSCC and provides potential targets for immunotherapy of HNSCC.

## 2. Materials and Methods

### 2.1. Identification of TILB-mRNAs

Microarray data (Affymetrix HG-U133_Plus 2.0 platform; Affymetrix, Santa Clara, CA, USA) from B cells and 18 other types of immune cells were downloaded from the GEO database, with the following accession numbers: GSE6863 (immature dendritic cells (DCs)), GSE8059 (natural killer (NK) cells and CD8^+^ T cells), GSE13906 (gamma–delta T cells), GSE23371 (immature DCs), GSE25320 (mastocytes), GSE27291 (gamma–delta T cells), GSE27838 (NK cells), GSE28490 (neutrophils, eosinophils, monocytes, B cells, NK cells, CD4^+^ T cells, CD8^+^ T cells, myeloid DCs (mDCs), and plasmacytoid DCs (pDCs)), GSE28726 (NK cells and CD4 T cells), GSE37750 (pDCs), GSE39889 (neutrophils), GSE42058 (mDCs), GSE49910 (B lymphocytes, CD4^+^ T cells, CD8^+^ T cells, central memory T cells, and monocytes), GSE51540 (T helper cell 17), and GSE59237 (DCs).

Raw data from the microarray datasets were background corrected, log2 transformed, and quantile normalized using the robust multiarray average (RMA) algorithm within the “affy” R packages [14, 15]. In total, 228 differentially expressed mRNAs were obtained through analysis of the expression differences of mRNAs between B cells and the 18 other types of immune cells, according to the standard of | log2 fold-change (FC) | >1 and *p* value < 0.05. Among the 228 B cell-related-mRNAs, only 180 mRNAs were found to be expressed in all patients with HNSCC in data from the TCGA (https://cancergenome.nih.gov/) and GEO databases, including GSE42743 (*n* = 103), GSE41613 (*n* = 97), and GSE65858 (*n* = 270), which were named as TILB-mRNAs. We used R packages “clusterProfiler,” “org.Hs.eg.db,” “enrichplot,” and “ggplot2” to conduct Gene Ontology (GO) enrichment analysis and Kyoto Encyclopedia of Genes and Genomes (KEGG) pathway analysis based on the TILB-mRNAs [16].

### 2.2. Construction of the TILB-Related Signature

Data from 546 patients with HNSCC were retrieved from the TCGA. After excluding patients who did not meet the criteria (survival time ≥ 30 days and clinical pathological characteristics), the RNA sequencing data and corresponding clinical information for 452 patients with HNSCC were downloaded and expression levels were standardized [17]. Data from the TCGA cohort were randomly divided into two groups as a training cohort (*n* = 226) and a testing cohort (*n* = 226), according to a 1:1 ratio. Univariate Cox proportional hazards analysis was used to identify survival-associated mRNAs among the TILB-mRNAs [18]. LASSO regression analysis was then performed for those TILB-mRNAs with *p*  < 0.01 in the univariate analysis. Finally, multivariable Cox regression analysis was used to construct the prognostic signature based on the results of LASSO regression, which was named the TILB-related signature. The risk score (RS) was defined as RS = ∑*β_i_* × *G*_*i*_, where *β* is the Cox regression coefficient of the mRNA and *G* is the mRNA expression value of gene *i*.

### 2.3. Validation of the TILB-Related Signature

Three RNA sequencing datasets of patients with HNSCC, including GSE42743 (*n* = 103), GSE41613 (*n* = 97), and GSE65858 (*n* = 270), were obtained from the GEO database as independent validation cohorts. Only 69, 96, and 267 patients with HNSCC in the three datasets, respectively, whose information met the criteria (survival time ≥30 days and clinical pathological characteristics) were used as three independent validation cohorts. Univariate and multivariate Cox regression analyses were performed on follow-up data to determine whether the RS was an independent factor affecting prognosis. Receiver operating characteristic (ROC) curve analysis was used to analyze the survival sensitivity and specificity of the model. Decision curve analysis (DCA) was used to compare differences in clinicopathological characteristics and RS prediction performance. Finally, we constructed a nomogram model. Calibration curves were used to test the accuracy of the predictive model for 1-, 3-, and 5-year OS. Patients were divided into high-risk and low-risk groups based on the median RS to verify the predictive performance of the model. A time-dependent ROC curve was constructed using the “survival ROC” R package, and Kaplan–Meier (K–M) survival curve analysis was performed using the “survival” R package; both curves were used to compare survival between the high and low-risk groups in the training cohort, testing cohort, total cohort, and the independent validation cohorts [19, 20].

### 2.4. Analysis of the Tumor Immune Microenvironment in the High- and Low-Risk Patient Groups

CIBERSORT (https://cibersortx.stanford.edu/), TIMER (https://cistrome.shinyapps.io/timer/), and microenvironment cell population (MCP) counters in the R package “Estimate” were used to analyze gene expression data [21–23]. Differences in immune-infiltrating cell populations between the high- and low-risk groups were analyzed to reveal the relationship between the RS and immune cell infiltration.

### 2.5. Analysis of the TILB-Related Signature Gene Expression

The tissue-specific expression levels of the genes in the prognostic signature were analyzed in the Human Protein Atlas (HPA; https://www.proteinatlas.org/) and their expression differences between cancer tissue and normal tissue was analyzed in the TCGA database.

### 2.6. Processing Clustering and Annotation of Single-Cell Sequencing Data

The data set GSE181919 was obtained from the GEO database, data for a total of 37 tissue samples were collected. Nontumoral surrounding normal tissue (*n* = 9) and primary cancer tissue (*n* = 20) were selected for analysis. The “Seurat” R package was used for filtering, using the criteria: cells with >200 genes, <8000 genes, <10% mitochondrial gene expression in the unique molecular identifier (UMI) count, and >0.1% gene expression. Batch effects of the samples were corrected using “RunHarmony” in the Harmony R package, and dimensionality reduction clustering was performed using the uniform manifold approximation and projection (UMAP) method (dims = 1:50). The cell type was determined according to the cell annotation of the dataset provider [24]. B cell subsets were identified by manually examining the expression levels of the canonical marker genes for B cells (*CD19*, *CD79A*, and *MS4A1*); plasma cells (*IGHG1*, *MZB1*, and *SDC1*); naïve B cells (*IGHD*, *FCER2*, *TCL1A*, and *IL4R*); memory B cells (*CD27*, *AIM2*, and *TNFRSF13B*); and GC B cells (*S1PI2*, *LRMP*, *SUGCT*, *MME*, *MKI67*, and *AICDA*) [25].

### 2.7. Statistical Analysis

R version 4.3.0 was used for the statistical analysis. Nonparametric tests were used for statistical tests between different groups and the log rank test was used to test for significant differences in survival probabilities between samples, with a *p*-value < 0.05 indicating statistical significance. Spearman rank correlation analysis was used to calculate the correlations.

## 3. Results

### 3.1. Identification of TILB-mRNAs

**Figure 1:**
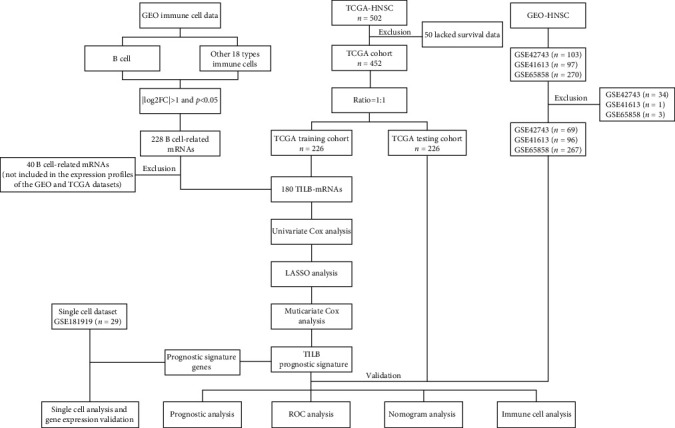
Flowchart of the study.

**Figure 2:**
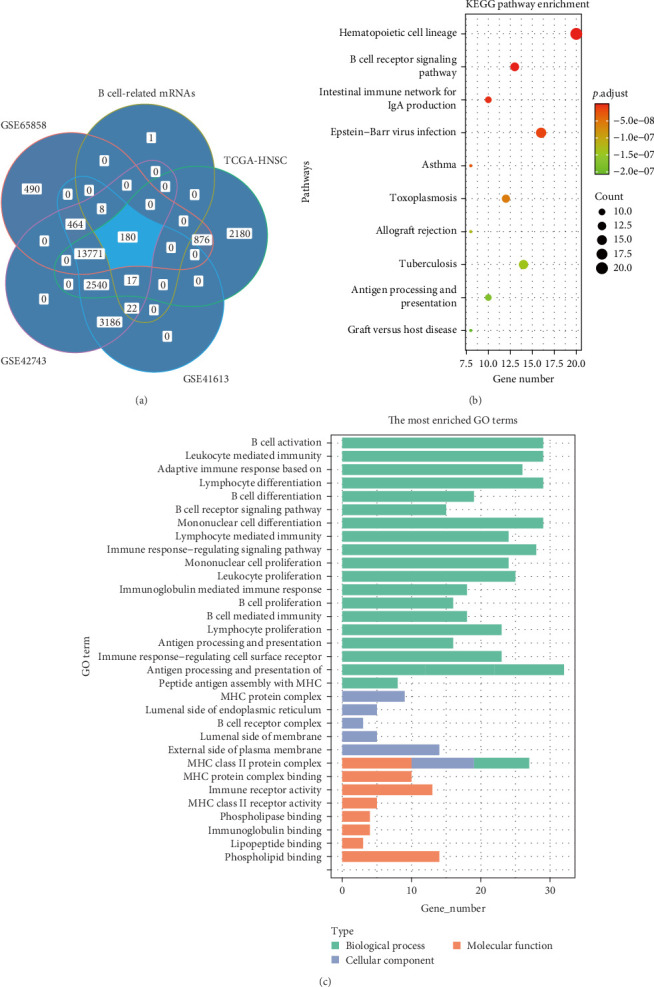
Tumor-infiltrating B cell (TILB)-mRNA extraction from RNA expression profiles and enrichment analysis. (A) Venn diagram of 228 differentially expressed mRNAs, 19,564 mRNAs from The Cancer Genome Atlas (TCGA)-HNSC, 15,789 mRNAs from GSE65858, and 20,188 mRNAs from GSE42743 and GSE41613. (B) Kyoto Encyclopedia of Genes and Genomes (KEGG) enrichment analysis of TILB-mRNAs. (C) Gene Ontology (GO) enrichment analysis of TILB-mRNAs.

The workflow of the computational steps is shown in a flowchart (Figure 1). Analyzing the differential gene expression between the acquired B cell data set and other immune cell data sets identified 228 B cell-related-mRNAs, which were then cross-referenced with mRNAs obtained from the expression profiles of patients with HNSCC from the TCGA and GEO databases (GSE42743, GSE41613, and GSE65858; Figure 2A). Consequently, 180 mRNAs were obtained and identified as TILB-mRNAs (Supporting Information 1: Table S1). Functional enrichment analysis showed that the TILB-mRNAs are characteristic of B cells, being enriched in biological process terms such as B cell activation, immunoglobulin mediated immune response, and B cell receptor signaling pathway (Figure 2B,C).

### 3.2. Identification of a TILB-Related Signature for Patients With HNSCC

**Figure 3:**
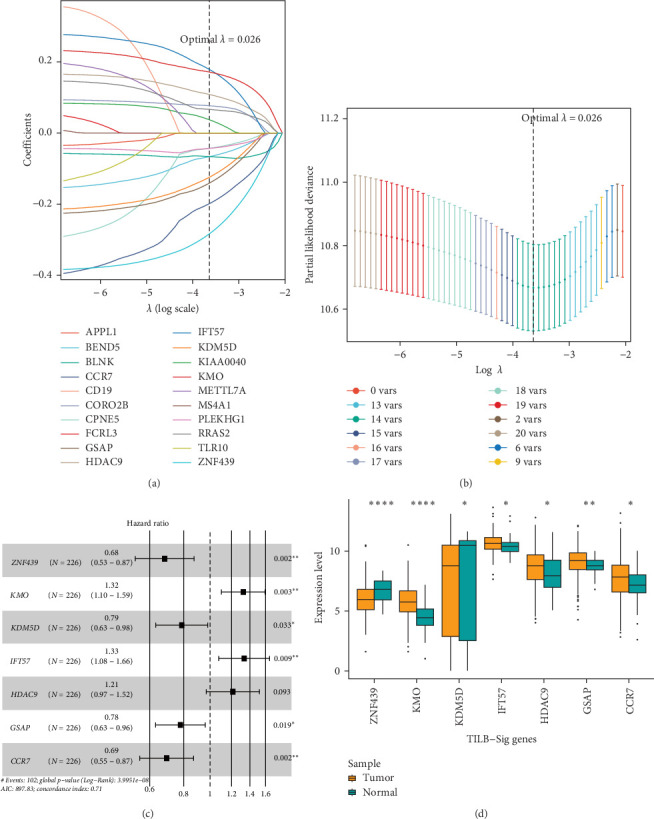
Acquisition of tumor-infiltrating B cell (TILB)-related signature. (A) Least absolute shrinkage and selection operator (LASSO) coefficient distribution of 20 mRNAs in the training cohort. (B) The coefficient profile generated according to the logarithmic *λ* sequence. Selection of the optimal parameter *λ* in the LASSO model. (C) LASSO regression analysis screening of forest maps of seven candidate B cell-related mRNAs associated with head and neck squamous cell carcinoma (HNSCC) survival and the construction of the prognostic signature. (D) Expression levels of signature mRNAs in tumor and normal tissues. *⁣*^*∗*^*p*  < 0.05; *⁣*^*∗∗*^*p*  < 0.01.

We randomly divided the TCGA cohort into training and test cohorts. In the training cohort, using univariate Cox regression analysis and LASSO regression analysis, seven mRNAs were identified as prognostic factors in patients with HNSCC (*ZNF439* (encoding zinc finger protein 439), *KMO* (encoding kynurenine 3-monooxygenase), *KDM5D* (encoding lysine demethylase 5D), *IFT57* (encoding intraflagellar transport 57), *HDAC9* (encoding histone deacetylase 9), *GSAP* (encoding gamma-secretase activating protein), and *CCR7* (encoding C–C motif chemokine receptor 7); Figure 3A,B). Multivariate analysis revealed that *ZNF439*, *KMO*, *KDM5D*, *IFT57*, *GSAP*, and *CCR7* were independent prognostic factors (Figure 3C). These mRNAs were then used to establish an mRNA prognostic signature. *ZNF439*, *KDM5D*, *GSAP*, and *CCR7* were identified as protective factors for the prognosis of patients with HNSCC, while *KMO*, *IFT57*, and *HDAC9* were identified as risk factors. The RS for prognosis was calculated as RS = 0.68 × Expi (*ZNF439*) + 1.32 × Expi (*KMO*) + 0.79 × Expi (*KDM5D*) + 1.33 × Expi (*IFT57*) + 1.21 × Expi (*HDAC9*) + 0.78 × Expi (*GSAP*) + 0.69 × Expi (*CCR7*), where Expi is the expression value of each mRNA. We analyzed the expression levels of the signature mRNAs in cancer tissues and normal tissues in the TCGA database. The results showed that *ZNF439* and *KDM5D* were highly expressed in normal tissues, whereas *KMO*, *FT57*, *HDAC9*, *GSAP*, and *CCR7* were highly expressed in tumor tissues (Figure 3D). We used the immunohistochemical data in the HPA database to analyze the expression differences of signature proteins between HNSCC tumor tissues and normal tissues. The results showed that *CCR7*, *KMO*, *IF57*, and *HDAC9* were highly expressed in HNSCC tumor tissues, while *ZNF439* was highly expressed in normal tissues (Supporting Information 2: Figure S1A–E). The above results showed that the signature mRNAs are related to the prognosis of patients with HNSCC.

### 3.3. Validation of the TILB-Related Signature for HNSCC

**Figure 4:**
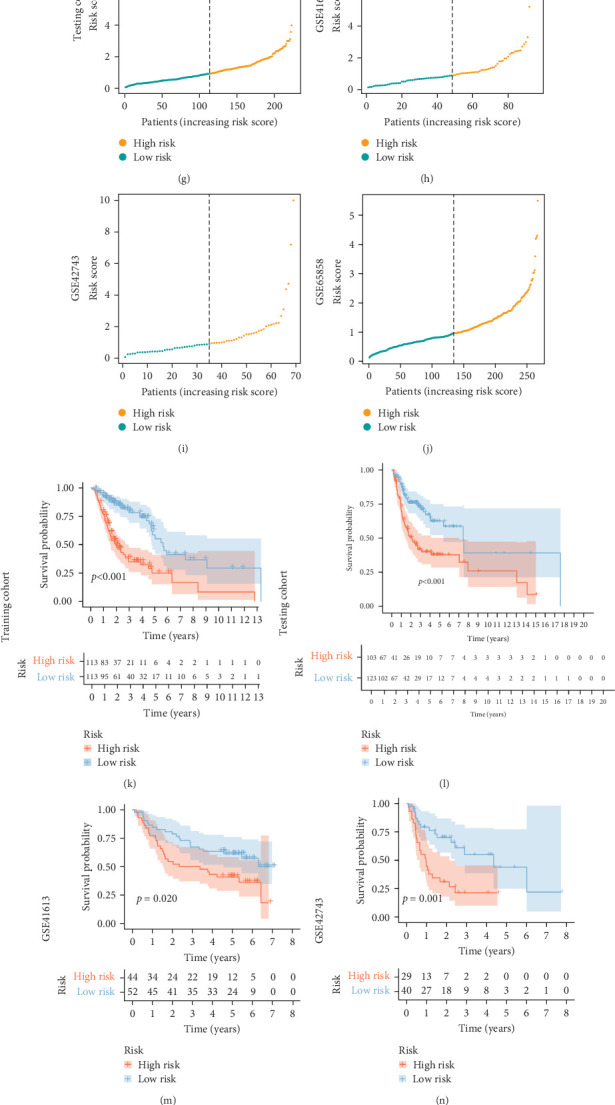
Construction and evaluation of the tumor-infiltrating B cell (TILB)-related signature. (A–J) TILB-related signature risk score (RS) analysis. The distribution of the scatter chart of the sample survival overview in the training cohort (A), the testing cohort (B), and the validation cohorts (GSE41613 (C), GSE42743 (D), and GSE65858 (E)). The distribution of the RSs in the training cohort (F), the testing cohort (G), and the validation cohorts (GSE41613 (H), GSE42743 (I), and GSE65858 (J)). Green dots and red dots denote survival and death, respectively. (K–O) Verification of the TILB-related signature. For the RS level of the model-based classifier, Kaplan–Meier (K–M) survival analysis was used to analyze the risk of death in the training cohort (K), the testing cohort (L), and the validation cohorts (GSE41613 (M), GSE42743 (N), and GSE65858 (O)) of head and neck squamous cell carcinoma (HNSCC; overall survival (OS) curves). (P–T) Time-dependent receiver operating characteristic (ROC) analysis of the sensitivity and specificity of survival for the TILB-related signature RS in the training cohort (P), the testing cohort (Q), and the validation cohorts (GSE41613 (R), GSE42743 (S), and GSE65858 (T)).

The median RS of the TILB-related signature was used to divide patients with HNSCC into high- and low-risk groups. The mortality rate in the low-risk group was lower than that in the high-risk group in the training, testing, and independent validation cohorts (Figure 4A–J), demonstrating that the observed mortality rate correlated with the RS. The results of K–M survival curve analysis showed that the OS of the low-risk group was longer than that of the high-risk group (*p*  < 0.001) in the training cohort (Figure 4K). The results in the testing cohort (*p*  < 0.005) and independent validation cohorts (*p*  < 0.05) were consistent with those of the training cohort (Figure 4), suggesting that the RS correlated with the OS time of patients with HNSCC. These results indicated that the good predictive value of the RS for the prognosis of patients with HNSCC. The quality of the signature was further evaluated by calculating the area under the curve (AUC) values, which were 0.682, 0.781, and 0.775 at the 1, 3, and 5-year follow-up time points, respectively, in the training cohort (Figure 4P) and 0.68, 0.666, and 0.622, respectively, in the testing cohort (Figure 4Q). The values were 0.627, 0.631, 0.67; 0.666, 0.669,0.772; and 0.574, 0.585, 0.669; respectively, in the independent validation cohorts (Figure 4). Hence, the TILB-related signature is a reliable measure of the prognostic risk in HNSCC.

### 3.4. The RS Is an Independent Prognostic Factor for Patients With HNSCC

**Figure 5:**
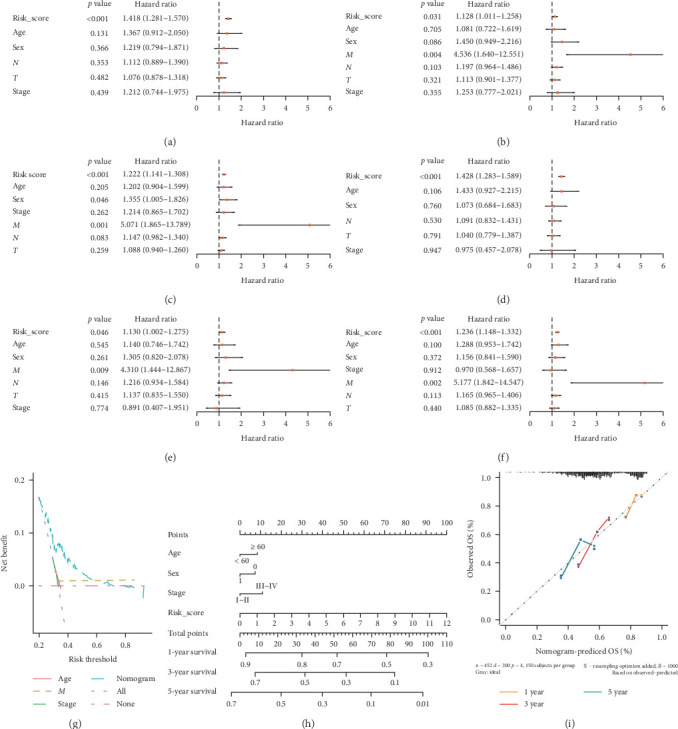
Risk score (RS) analysis and nomogram construction to evaluate of overall survival (OS) of patients with head and neck squamous cell carcinoma (HNSCC). (A–C) The independent prognostic value of the RS was evaluated using Cox regression analysis. Univariate Cox regression analysis of models in the training cohort (A), the testing cohort (B), and the total The Cancer Genome Atlas (TCGA) cohort (C). (D–F) Multivariate Cox regression analysis of models in the training cohort (D), the testing cohort, (E), and the total TCGA cohort (F). (G) Decision curve analysis (DCA) analysis of the ability of the RS and other clinicopathological factors to predict the OS of patients with HNSCC. (H) Nomogram to predict the OS rate of patients with HNSCC. (I) Nomogram calibration chart during 1, 3, and 5 years of follow-up.

To further evaluate the RS as an independent prognostic marker in patients with HNSCC, univariate COX regression analysis in the survival ROC R package was used to compare the RS with other clinicopathological factors (age, sex, tumor size (*T*), lymph node metastasis (*N*), distant metastasis (*M*), and clinical stage; Figure 5A–F). Multivariate COX regression analysis showed that the RS was associated with OS (Figure 5D), with *p* values < 0.05 in the training, testing (Figure 5D,E), and total cohorts (Figure 5F). This indicated that the RS was a significant independent prognostic factor for HNSCC, independent of clinicopathological parameters. The sensitivity and specificity of the RS in predicting the prognosis of patients with HNSCC was investigated by comparing the area under the DCA (Figure 5G), which measured changes in the RS and other clinicopathological factors in predicting the OS of patients with HNSCC. The RS showed a better predictive value than the other clinicopathological factors, indicating that it is more effective to predict HNSCC prognosis. To develop a clinically applicable method to predict the survival probability of patients, we generated a nomogram using the rms R package (Figure 5H), plotting RS, age, stage, and sex. The RS made the largest contribution to the 3- and 5-year OS of patients with HNSCC. In addition, we supplemented our model with the 1-, 3- and 5-year calibration charts. The 1-, 3- and 5-year OS calibration curves showed good prediction compared with the ideal models in all cohorts (Figure 5I). The results showed that the nomogram could independently evaluate the survival of patients with HNSCC, which might help clinicians to make better medical decisions and improve follow-up plans.

### 3.5. Differences in the Tumor Immune Microenvironment Between the Low- and High-Risk HNSCC Groups

**Figure 6:**
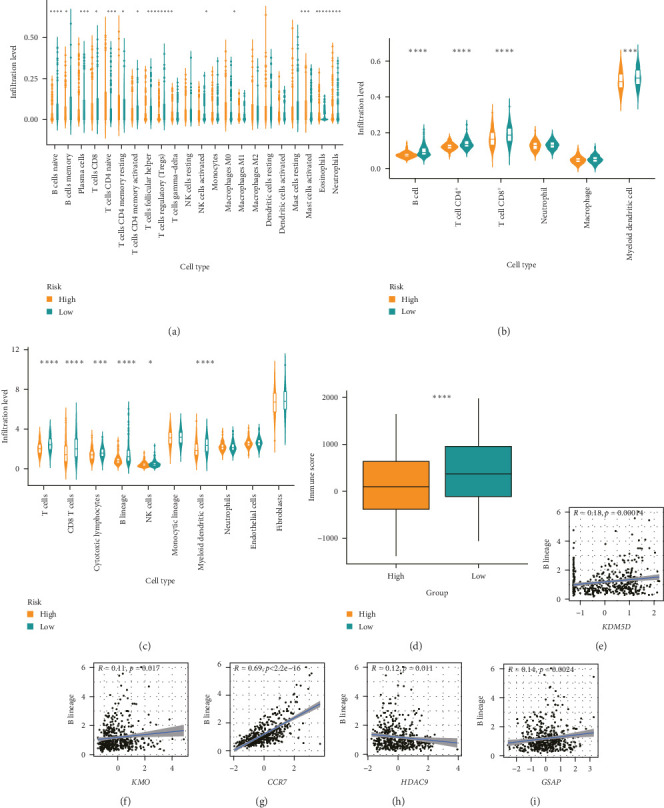
Computational analysis of immune cell infiltration in patients with head and neck squamous cell carcinoma (HNSCC). (A–C) The differences in the numbers of infiltrating immune cells between the high-risk group and the low-risk group. *⁣*^*∗*^*p*  < 0.05, *⁣*^*∗∗*^*p*  < 0.01, *⁣*^*∗∗∗*^*p*  < 0.001, *⁣*^*∗∗∗∗*^*p*  < 0.0001. (A) Immune cell infiltration analysis results produced by “CIBERSORT,” (B) immune cell infiltration analysis results produced by “TIMER,” (C) immune cell infiltration analysis results produced by “microenvironment cell population (MCP) counter.” (D) Immune cell infiltration score analysis. (E–I) Correlation analysis of model genes and B cell infiltration.

To explore the differences in immune cell infiltration between the high- and low-risk groups, the infiltrating immune cells in the HNSCC microenvironment were analyzed using CIBERSORT, TIMER, and MCP counter (Figure 6A–C). In the CIBERSORT analysis, genes related to the naive B cells, memory B cells, plasma cells, CD8^+^ T cells, CD4^+^ memory activated T cells, follicular helper T cells, resting mast cells, and activated mast cells were found to be expressed at higher levels in the low-risk group than in the high-risk group. In addition, the expression levels of genes related to regulatory T cells (Tregs), CD4^+^ naive T cells, CD4^+^ memory resting T cells, activated NK cells, activated mast cells, and eosinophils were significantly lower in the low-risk group than in the high-risk group. In the TIMER and MCP counter analyses, the degree of infiltration of B cells, CD4^+^ T cells, CD8^+^ T cells, mDCs, and cytotoxic lymphocytes was higher in the low-risk group than in the high-risk group. The low-risk group had a better overall immune response than the high-risk group (Figure 6D). These results suggested that the prognostic signature is capable of distinguishing the tumor microenvironment in the low and high-risk groups of the HNSCC population. Correlation analysis showed that *CCR7*, *KMO*, *GSAP*, and *KDM5D* correlated positively with the degree of B cell infiltration, while *HDAC9* correlated negatively with B cell infiltration (Figure 6).

### 3.6. Analysis of TILB-Related Signature Genes in Single-Cell Sequencing Data

**Figure 7:**
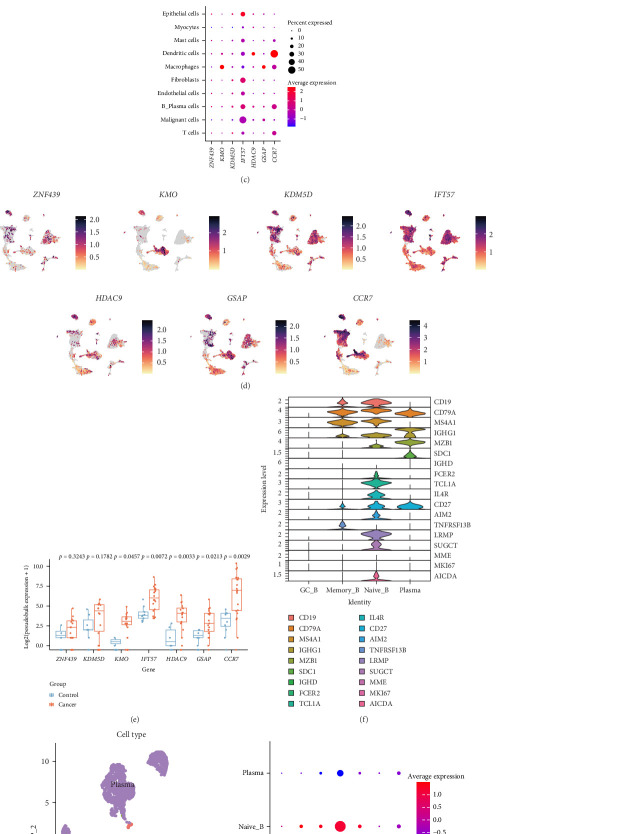
Single-cell sequencing data analysis. (A) Annotation of cell types in the clusters. (B) Proportions of different cells in cancer tissue and normal tissue. (C) Expression levels of signature genes in different cells. (D) Uniform manifold approximation and projection (UMAP) analysis showing expression of signature genes in subtypes of cancer cells. (E) Expression levels of the tumor-infiltrating B cell (TILB)-related signature genes in B Plasma cells. (F) Expression levels of marker genes in different subtypes of B Plasma cells. (G) Subtypes of B Plasma cells. (H) Expression levels of signature genes in different subtypes of B Plasma cells.

Based on the expression of marker genes provided by the single-cell dataset, 10 different cell populations were annotated, namely, B plasma cells, DCs, endothelial cells, epithelial cells, fibroblasts, macrophages, malignant cells, mast cells, myocytes, and T cells (Figure 7A). We found that B cell infiltration in tumor tissue was higher than that in normal tissue (Figure 7B). In addition, the proportions of T cells, fibroblasts, macrophages, mast cells, and DCs were also higher in tumor tissue than in normal tissue. However, macrophages accounted for a relatively high proportion in normal tissue. At the same time, the results showed that the TILB-related signature genes were expressed in B cells and plasma cells (Figure 7C,D). Analysis of the expression of the TILB-related signature genes in B Plasma cells showed that *KMO*, *IFT57*, *HDAC9*, *GSAP*, and *CCR7* were significantly highly expressed in tumor tissue, while *ZNF439* and *KDM5D* showed a similar trend. (Figure 7E). In addition, a subset of B Plasma cells was extracted for repopulation and four different B cell subsets were annotated based on the expression of the marker genes, namely, GC B cells, memory B cells, naïve B cells, and plasma cells (Figure 7F,G). We found that *IFN57* and *CCR7* were highly expressed in naïve B cells and memory B cells, respectively (Figure 7H). In conclusion, the TILB-related signature genes are expressed in B cells, and there are differences in their expression among different cell subtypes.

## 4. Discussion

Studies have suggested that TILs are associated with tumor progression, prognosis, and treatment response. In recent years, immune cell infiltration analysis, based on transcriptome data, has shown that TILs have characteristic patterns, opening up new methods for evaluating TILs based on gene expression data [13, 26]. B cells are one of the main components of tumor-infiltrating immune cells, and recently, TILBs have been suggested to play an important role in tumor immunity and therapy [27, 28]. Although the T cell-related immune response has become a therapeutic target [29], low response rates, adaptive/acquired resistance, and adverse reactions still prevent most patients with cancer from obtaining a sustained clinical benefit. For these patients, B cell-related treatments might represent a breakthrough. Recently, Ruffin et al. [8] reported that the presence of GC TILBs in the TLS is associated with better prognosis in patients with HNSCC. Concomitantly, poor control of head and neck tumors was observed in mice in which peripheral B cells had been depleted [30]. In conclusion, B cells play an important role in the control of head and neck tumors. Therefore, we need to find new markers and characteristics to evaluate TILBs and carry out prognostic stratification in patients with HNSCC.

The goal of this study was to assess the degree of infiltration of TILBs in HNSCC and to construct a TILB-related signature for HNSCC prognosis. The prognostic value of the TILB-related signature was confirmed and validated by rigorous computational assessment, including K–M analysis, ROC analyses, and multivariate Cox regression analysis. The prognostic signature could help to provide a more personalized risk assessment for HNSCC treatment. The TILB-related signature could reflect the degree of B cell infiltration in tumor tissues and could further identify high-risk patients with HNSCC with poor survival outcomes.

Our research showed *KDM5D* and *CCR7* are protective factors for the prognosis of patients with HNSCC, while *KMO*, *IFT57*, and *HDAC9* are risk factors. These results are consistent with previous research. Schäfer et al. [31] found that *KDM5D* can increase the sensitivity of patients with cancer to chemotherapeutic drugs. In addition, *KDM5D* can reduce malignant gene expression and inhibit the development of various cancers [32, 33]. Wang et al.'s [34] findings indicate that *KDM5D*, a key marker of immune infiltration in laryngeal squamous cell carcinoma, correlates with extended OS in patients with high expression levels. *CCR7* is a chemokine receptor and its role in tumors is controversial. Some studies have shown that *CCR7* promotes tumor migration [35]. However, some studies have shown that high expression of *CCR7* improves the prognosis of patients with head and neck cancer [36]. Immune cells expressing the chemokine receptor *CCR7* migrate to the TLS through lymphatic vessels via the chemokine *CCL21*, playing an important role in the formation of the TLS [37]. *HDAC9* is a poor prognostic factor in malignant tumors [38, 39]. In head and neck malignant tumors, overexpression of *HDAC9* promotes carcinogenesis by targeting the transcription factor *MEF2D* and the proapoptotic *MEF2* target, *NR4A1*/*Nur77* [40, 41]. At the same time, upregulation of *HDAC9* might be the cause of the inherent drug resistance of tumor cells [42]. KMO is a key enzyme in tryptophan metabolism. Upregulation of KMO increases the level of quinolinic acid, which stimulates NMDA receptors to initiate gene activation and cell proliferation, thereby, promoting breast tumor cell survival [43]. Besides, the nonenzymatic function of KMO can promote the stability of *β*-catenin and the expression of pluripotency genes, thereby, promoting the proliferation, survival, invasion, and metastasis of tumor cells [44]. *IFT57* exerts an oncogenic role in non-small cell lung cancer by activating DNA replication and cell cycle pathways via upregulation of MCM2, MCM6, and MCM8 expression [45]. The above studies support the results of our research on the prognostic effects of the genes constituting the prognostic model.

Immune cell infiltration analysis showed that the infiltration levels of Tregs, naive B cells, memory B cells, plasma cells, CD8^+^ T cells, CD4^+^ memory activated T cells, follicular helper T cells, and mast cells in the low-risk group were higher than those in the high-risk groups. Meanwhile, tumor-promoting cells, such as naive CD4^+^ T cells, macrophage Mo cells, activated mast cells, and resting CD4^+^ T cells were highly infiltrated in the high-risk group. This finding is consistent with a T cell-mediated antitumor response. Activated CD4^+^ T cells can reconstitute the immune microenvironment and promote tumor clearance [46]. In addition, CD8^+^ T cells are the main drivers of the antitumor effect [47]. Our study found that increased infiltration of TILBs was associated with good prognosis, and TILBs might promote antitumor immunity in multiple ways, including presentation of tumor antigens to CD4^+^ T cells [48, 49] or enhancing antitumor immunity by producing tumor-specific antibodies [50, 51]. The function of TILBs in the TLS is associated with increased survival and response to immunotherapy in patients with cancer. Gaglia et al. [52] identified a cellular network of direct interactions between tumor-infiltrating cells, termed lymphonets, in which B cells favor the maintenance of stem-like CD8^+^ T cell populations within tumors. In this study, the degree of Treg infiltration in the low-risk group was higher than that in the high-risk group, which was associated with good prognosis. In general, the infiltration of Tregs is associated with poor prognosis of patients with tumors. However, some studies have shown that FoxP3^+^ Treg infiltration is beneficial to the prognosis of patients with HNSCC [53, 54]. These observations might be related to the differences in tumor types, the diversity of molecular features, the heterogeneity of Tregs, their distribution, and their role in the infiltration of certain immune subpopulations in tumors.

Single-cell sequencing is a powerful approach to study tumor specificity and clustering [55]. Single-cell analysis revealed that the infiltration of B plasma cells, macrophages, DCs, and mast cells in HNSCC was higher than that in normal tissues, which is consistent with the general characteristics of high immune infiltration in head and neck tumors. By contrast, fibroblasts, endothelial cells, myocytes, and other cells account for a higher proportion in normal tissues than in HNSCC. Among the TILB-related signature genes, *IFT57* and *CCR7* are highly expressed in immune cells, which might be related to the important role of IFT57 in the formation of immune synapses and the chemotactic function of CCR7 on immune cells. We found that *IFT57* was highly expressed in naïve B cells, while *CCR7* was highly expressed in memory B cells. This relationship might be related to gene function in B cell subsets [56, 57]. The results confirmed that TILB-related signature genes are highly expressed in B cells and other immune cells, but are differentiated among B cell subtypes, which might be related to the function of the encoded proteins.

In addition, the expression of TILB-related signature genes in B cells of tumor tissues was higher than that in B cells of normal tissue, which correlated with the important role of B cells in antitumor and inflammatory responses in head and neck tumors. Compared with the results of differential expression analysis in the TCGA, the expression trends of *ZNF439* and *KDM5D* were opposite, which is related to the different expression levels of genes in whole tissues and in pure B cells. The function of TILB-related signature genes in different B cell subtypes awaits further study.

In this study, a TILB-related signature for HNSCC was constructed and validated, which could be used as a prognostic tool, independently of other clinical and pathological features. This signature provides a new method to monitor TILBs. This signature could reflect the changes of TILBs from different aspects and could support rational diagnosis and individualized treatment. Although this study provides new insights into the development of novel therapies for HNSCC, several limitations remain. For example, further validation in prospective cohort studies is needed. In addition, the limited number of single cell RNA sequencing samples and the limited amount of data published in public databases might represent sources of bias. In summary, this study provides a new method to predict the prognosis of patients with HNSCC, and the identified TILB genes might provide new targets for future immunotherapy.

## Data Availability

The datasets presented in this study can be found in online repositories. The names of the repositories and accession numbers can be found in the article.

## Supporting Information

Supporting Information 1 Table S1: provides information on TILB-mRNAs.

Supporting Information 2 Figure S1: in the supporting information for a comparison of the expression of model genes in tumor tissue and normal tissue.
