# Supplementary material for: Identification of a Prognostic Signature Based on Tumor-Infiltrating B Lymphocyte mRNA in Head and Neck Squamous Cell Carcinoma
Source: J Immunol Res. 2025 Mar 19;2025:9375885. doi: 10.1155/jimr/9375885 (PMC11944952; doi:10.1155/jimr/9375885)
Supplement: Supporting Information 1 — Table S1: provides information on TILB-mRNAs. [file 9375885.f1.docx]

TILB-mRNAs

ZNF92

ZNF573

ZNF532

ZNF439

ZCCHC7

ZCCHC18

WDFY4

VPREB3

UCP2

TXNIP

TSPAN3

TSPAN13

TRAF5

TPD52

TNFRSF17

TNFRSF13B

TMEM156

TLR10

TLE1

TCL1A

TCF4

TBC1D9

SYT17

SYPL1

SYK

SYBU

SWAP70

STX7

STRBP

STAP1

STAG3

ST6GAL1

SSPN

SP140

SNX29

SNX22

SNX2

SMAGP

SLC38A11

SLC15A2

SLAMF6

SGCE

SETBP1

SESTD1

SEMA4B

SELL

SEL1L3

SAV1

RRAS2

RPS5

RPS4Y1

RPS27

RPS19

RNASE6

RHOH

RASGRP3

RASEF

RALGPS2

QRSL1

PTPRK

PRICKLE1

PPM1K

POU2AF1

PNOC

PLEKHG1

PLEKHF2

PLAC8

PKIG

PKHD1L1

PDLIM1

PCDH9

P2RY10

P2RX5

MTSS1

MS4A1

MILR1

METTL8

METTL7A

MEF2C

MARCKS

MAP3K1

LY86

LTB

LPAR5

LBH

LAT2

KMO

KIAA0040

KDM5D

KCNH8

JAM3

JADE3

ITPR1

ISG20

IRF8

IL7

IL4R

IKZF3

IFT57

ID3

HVCN1

HLA-DRA

HLA-DQA1

HLA-DPB1

HLA-DPA1

HLA-DOB

HLA-DOA

HLA-DMB

HLA-DMA

HHEX

HDAC9

HBB

GSAP

GPR18

GNG7

GNG11

GAPT

FCRLA

FCRL5

FCRL3

FCRL2

FCHSD2

FCGR2B

FCER2

FAM3C

ETS1

EML6

EIF1AY

EBF1

EAF2

E2F5

DSP

DRAM2

DOK3

DAPP1

CYSLTR1

CXXC5

CTSH

CR2

CR1

CPNE5

CORO2B

COBLL1

CNTNAP2

CLIC4

CLECL1

CIITA

CDCA7L

CD79B

CD79A

CD74

CD72

CD52

CD37

CD24

CD22

CD200

CD1D

CD1C

CD19

CD180

CCR7

CCR6

CCDC50

BTLA

BTK

BLNK

BIRC3

BEND5

BCL7A

BCL2

BCL11A

BANK1

BACE2

ARHGAP24

APPL1

ALOX5

AIM2

AFF3

ABCB4
