# Supplementary material for: Identification of a Prognostic Signature Based on Tumor-Infiltrating B Lymphocyte mRNA in Head and Neck Squamous Cell Carcinoma
Source: J Immunol Res. 2025 Mar 19;2025:9375885. doi: 10.1155/jimr/9375885 (PMC11944952; doi:10.1155/jimr/9375885)
Supplement: Supporting Information 2 — Figure S1: in the supporting information for a comparison of the expression of model genes in tumor tissue and normal tissue. [file 9375885.f2.pdf]

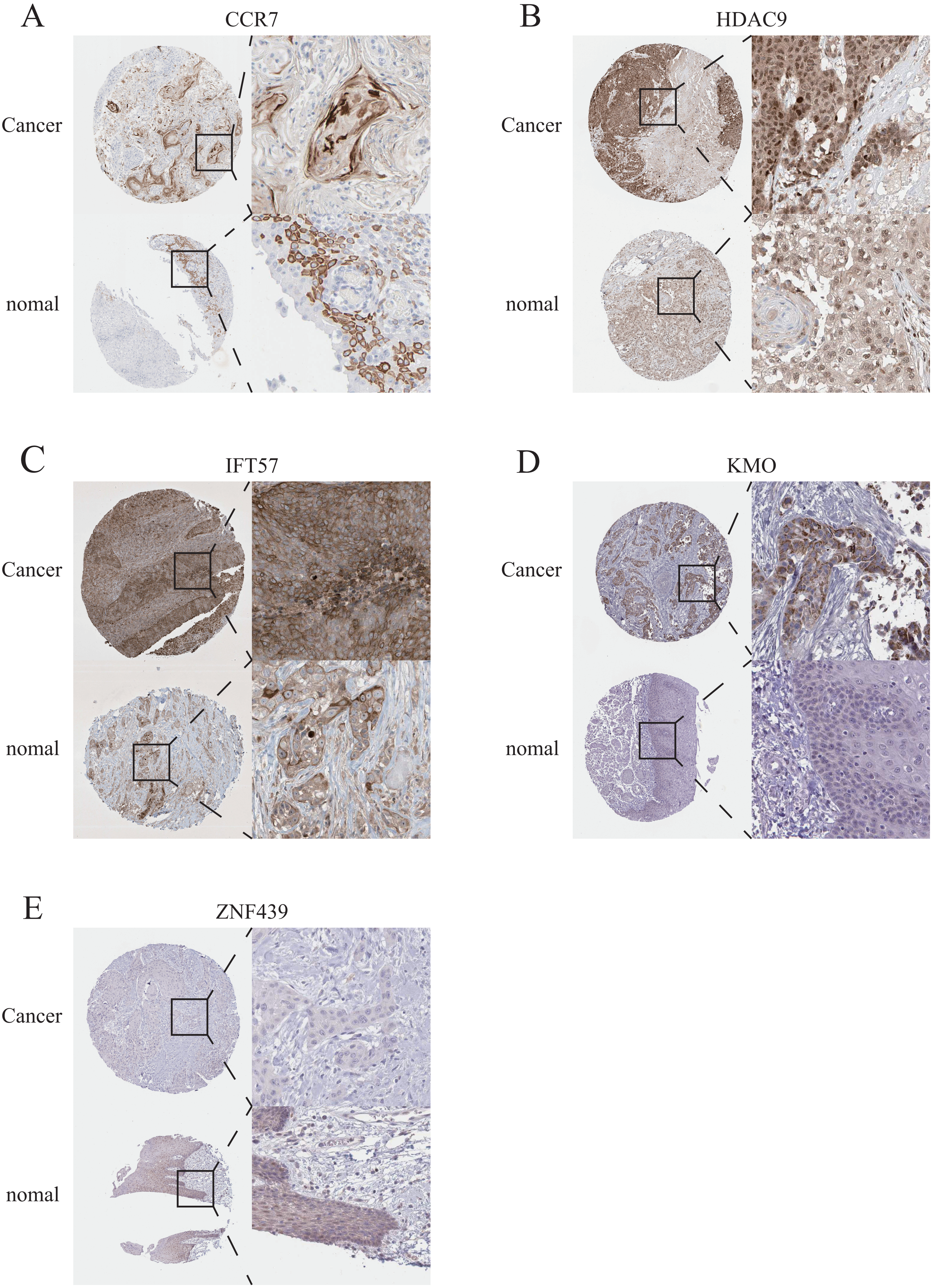

A
CCR7
Cancer
nomal
B
HDAC9
Cancer
nomal
C
IFT57
Cancer
nomal
D
KMO
Cancer
nomal
E
ZNF439
Cancer
nomal
